# Prevalence of Cervical Cancer Screening and Awareness among Women in an Urban Community in South India—A Cross Sectional Study

**DOI:** 10.5334/aogh.2735

**Published:** 2020-03-16

**Authors:** Alyse Reichheld, Pavan Kumar Mukherjee, Sajitha MF Rahman, Kirubah V. David, Ruby Angeline Pricilla

**Affiliations:** 1 Tufts University School of Medicine, Boston, Massachusetts, US; 2 Department of Medical Social Works, Low Cost Effective Care Unit, Christian Medical College, Vellore, Tamil Nadu, IN; 3 Department of Family Medicine, Low Cost Effective Care Unit, Christian Medical College, Vellore, Tamil Nadu, IN; 4 Department of Community Medicine, Low Cost Effective Care Unit, Christian Medical College, Vellore, Tamil Nadu, IN

## Abstract

**Background:**

Although the incidence of cervical cancer has declined in developed countries, cervical cancer remains a major problem in those that are developing. Past studies suggest that Indian women, who account for at least one-fourth of the global disease burden, are not routinely screened.

**Objectives:**

Among the women living in our low-income urban community in South India, we sought to determine the prevalence of screening and to assess women’s knowledge of cervical cancer.

**Methods:**

We conducted a community-based cross-sectional survey evaluating cancer screening prevalence among women aged 25–65 living in the communities served by our clinic. We also assessed knowledge of cervical cancer, screening and the HPV vaccine in a subset of 175 women in the same age range.

**Findings:**

Prevalence data was available for 1033 women. Of these,14.3% had at least one lifetime pelvic exam and 7.1% had undergone cervical cancer screening. Women who were married below the age of 18, who belonged to non-Hindu religion, and who were from a higher socioeconomic status were more likely to be screened. Women who were single did not undergo screening. With regard to knowledge of cervical cancer, 84.6% of women had poor knowledge, 10.3% had moderate knowledge, and 5.1% had good knowledge. Women aged 41 years or younger had better knowledge of the disease.

**Conclusions:**

Very few women are screened for cervical cancer and few have adequate knowledge of the disease within this South Indian community. These findings suggest opportunities for a community-based education and screening campaign to reduce the prevalence of cervical cancer within this population.

## Introduction

The global cervical cancer burden is disproportionally high in low and middle-income countries, where 83% of all new cases and 85% of cervical cancer deaths occur [1]. India accounts for nearly one-fourth of the world’s cervical cancer deaths, with 60,078 deaths and 96,922 new cases in 2018 [2,3]. This largely preventable disease is the second most common cause of cancer mortality among Indian women [4]. Through Human Papilloma Virus (HPV) vaccination and screening campaigns, higher income countries have successfully reduced their burden of cervical cancer by as much as 65% over four decades [5]. Vaccination of adolescents against HPV 16 and HPV 18, which cause approximately 70% of cervical cancers, can prevent the majority of cervical cancer cases [3]. Additionally, frequent screening allows for early detection and removal of precancerous lesions.

In spite of these successful prevention methods, one study reported that lifetime screening prevalence for Indian women ages 15 to 49 was only 29.8% in India [6]. In addition to the lack of a national screening campaign for cervical cancer, several studies have shown that knowledge of cervical cancer, prevention, and screening are limited among women across different settings in India [7,8,9,10].

Currently, there is no available data regarding screening for women living in our urban community. The aim of this study was two-fold: We sought to determine the prevalence of cervical cancer screening among women aged 25–65 living in a low-income urban community, and within this subset of women, we assessed their baseline knowledge and awareness of cervical cancer, screening, and the HPV vaccine. The findings from this study will help us better understand potential opportunities for education and screening.

## Materials and Methods

### Setting and Participants

This community-based cross-sectional study was conducted among women ages 25–65 who live in the low-income urban population of Vellore, Tamil Nadu. The Low Cost Effective Care Unit provides care to these five communities, a population of 10,000 patients, through community health workers. The community health workers act as the interface between the community and the main clinic through weekly outreach clinics, which occur in community spaces. Prevalence and knowledge data were collected at these outreach clinics as well as via home visits. This study was conducted from June to July of 2019.

### Prevalence Survey

Demographic data and screening prevalence was acquired from women ages 25–65 living in the community. Details of screening were obtained from community health workers as part of their scheduled work at the outreach clinics. This survey included demographic data (age, education level, occupation, religion, socioeconomic status, parity, age of marriage, age at birth of first child). Additionally, women were asked whether they had undergone a pelvic exam, which screening tests were performed, including Visual Inspection with Acetic Acid (VIA), Visual Inspection with Lugdol’s Iodine (VILI), and Cytology. Women were also asked for the results of the tests. In Tamil Nadu, VIA/VILI are performed at government hospitals, while the Low Cost Effective Care Unity and Christian Medical College have the capacity to provide cytology testing. Finally, women were asked whether they were willing to be screened.

### Knowledge Survey

In a subset of 175 women, we also conducted a ten-question survey regarding women’s knowledge of cervical cancer, screening, and the HPV vaccination. We determined that a sample size of 175 for the knowledge survey was adequate based on a study in Andhra Pradesh [10], which reported that 38% of surveyed women had good awareness regarding cervical cancer, and a relative precision rate of 20%. A research assistant, with the help of a translator, conducted the semi-structured survey. Researched obtained written informed consent from each participant.

The survey to assess women’s knowledge of cervical cancer was adapted and modified from a study done in an Andhra Pradesh hospital [10]. Our survey had two parts. The first part included details regarding demographic factors (age, education level, occupation, religion, socioeconomic status, parity, age of marriage, and age at birth of first child) and HPV vaccination status. In the second part of the survey, knowledge regarding cervical cancer symptoms, signs, risk factors, prevention, screening, treatment, and HPV vaccination was assessed using a 15-point scale.

Participants were awarded one point if they knew about cervical cancer. They were given two points for any two correct responses for symptoms associated with cervical cancer (such as bleeding between periods, foul smelling discharge, bleeding after intercourse, postmenopausal bleeding, urinary urgency, severe back ache and lower abdominal point, and severe swelling in one of both legs and feet), risk factors for developing cervical cancer (such as having multiple sex partners, early sexual intercourse, acquiring HPV, cigarette smoking, young age at first birth, use of oral contraceptive for over five years, history of sexually transmitted diseases, poor menstrual hygiene, and more than five pregnancies), prevention methods (including avoid multiple sexual partners, avoid early sexual intercourse, vaccinate against HPV, quit smoking, avoid birth at early age, and avoid usage of oral contraceptives), and treatment for cervical cancer (such as drug therapy, radiotherapy, and surgery).

In the final part of the survey, women were asked questions to determine their knowledge of screening. Participants were given one point for each correct answer regarding types of screening tests (VIA, VILI, pap smear), eligibility for screening (women age 25 years and above, women with multiple sex partners, elderly women), location of screening (private and public hospitals) and frequency of screening (once every three years or once every five years). Two points were awarded if the participant knew details about the HPV vaccination. The maximum possible score was fifteen and minimum score was zero.

Participants were then categorized based on their correct responses; a total score greater than or equal to 12 was considered good knowledge, 8–11 points implied moderate knowledge, and a score less than 8 points meant poor knowledge.

### Data analysis

Responses for each knowledge survey question were recorded in Epidata (Version 3.1) and prevalence data were recorded in Microsoft Excel 2010. Both data were analyzed using SPSS 23. Descriptive statistics were calculated, including proportions for categorical variables and means (SD) for continuous variables. We used uni-variate analysis to determine the prevalence of cervical cancer screening, prevalence of positive test results, and treatment in this patient population. Chi-square tests were used to find if there is a significant association between age, religion, education, occupation, SES, parity and marital status with screening for cervical cancer.

Measures of central tendency and standard deviation were calculated for the aggregate scores on knowledge. We examined the relationship between knowledge of cervical cancer screening and demographic data using chi-squared tests. A p-value of <0.05 was considered as significant.

## Results

### Screening Prevalence

#### Demographic Data

There are 2,514 women aged 25–65 years in the population served by the urban health center. Among them, data regarding screening was available for 1,033 (41.1%) women (Table 1). Our participants were evenly distributed across age groups. The mean (SD) age of women was 41.8 (11.5) years. The median was 40 years. More than one-fourth (26.4%) of women had a high school education and 5.5% attended college. The mean (SD) education level was 5.8 (4.7) years. The median years of education was six years and the range included zero years (no education) to nineteen years of education. The majority of women belonged to the Hindu religion (871, 84.3%), were married (802; 77.6%), and were homemakers (641; 62.1%). The mean age of marriage was 19.0 (3.6) years and the mean age of first childbirth was 20.6 years (3.7). On average, women had 2.5 children with a standard deviation of 1.4 children.

**Table 1:** Social demographic distribution of the study participants (n = 1033).

Category	Range	Number (%)

Age	25–34	333 (32.2)
	35–44	270 (26.2)
	45–54	239 (23.1)
	55–64	191(18.5)
Religion	Hindu	871 (84.3)
	Christian	129 (12.5)
	Others	33 (3.2)
Education	Nil	293 (28.4)
	1–5 years	219 (21.2)
	6–8 years	191 (18.5)
	9–12 years	273 (26.4)
	College	57 (5.5)
Occupation	Housewife/household work	641 (62.1)
	Unskilled	228 (22.1)
	Semi-skilled	30 (2.9)
	Skilled	45 (4.4)
	Clerical	2 (0.2)
	Small Business owner	66 (8.4)
	Professional	21 (2.0)
Socio-Economic Status	Lower	727 (70.4)
	Middle	303 (29.3)
	Upper	3 (0.3)
Marital Status	Married	802 (77.6)
	Widow	174 (16.8)
	Separated/Divorced	17 (1.6)
	Single	40 (4.0)
Age of Marriage (n = 993)	<18	329 (33.1)
	≥18	664 (66.9)
Age of First Child (n = 937)	≤20	543 (58.0)
	>20	394 (42.0)
Parity	No Children	96 (9.3)
	1–2	430 (41.7)
	>2	507 (49.0)

#### Screening data

One hundred and forty eight women (14.3%) had a pelvic exam in their lifetime. Twenty-four (2.3%) women had undergone hysterectomies. Forty-nine (4.7%) women had cytology and three (0.3%) women had cytology twice. Among them, 42 samples were negative, five were unsatisfactory for cytology, and one was positive. The positive test underwent a colposcopy, which was negative. Twenty-eight (2.7%) women had VIA/VILI. Twenty-three were negative, three were positive, and one result was not available. The three positive tests were referred to a higher center. Four women had VIA/VILI testing as well as cytology. No single women were screened and five of the women who had no children were screened. Excluding women who had hysterectomies, 64.6% (652) women were willing to undergo screening for cervical cancer.

Women who were married below 18 years of age were more likely to be screened (p-value = 0.01, OR = 1.85, 95% CI = 1.14–2.99). Additionally, women of a higher socioeconomic status (p-value-0.006, OR = 0.51, 95% CI = 0.32–0.83) and those belonging to the non-Hindu religion (p = 0.00, OR = 0.39, 95% CI = 0.23–0.67) were more likely to be screened. There was no significant association between age, education, occupation, marital status, age of first childbirth and parity with screening (Table 2).

**Table 2:** Factors influencing screening.

Category	Screening, n (%)		Chi square	p-value	Odds Ratio (95% CI)

	Yes	No			

**Age** (n =1033)					
≤40	30 (5.7)	494 (94.3)	2.92	0.08	0.66 (0.41–1.07)
>40	43 (8.4)	466 (91.6)			
**Religion** (n = 1033)						
Hindu	51 (5.9)	820 (94.1)	**12.4**	**0.00**	**0.40 (0.23–0.67)**
Others	22 (13.6)	140 (86.4)			
**Education** (n = 1033)					
Less than high school	51 (7.3)	652 (92.7)	0.12	0.73	1.10 (0.65–1.83)
High school and above	22 (6.7)	308 (93.3)			
**Occupation** (n = 1033)					
Housewife	48 (7.5)	593 (92.5)	0.46	0.5	1.19 (0.72–1.96)
Others	25 (6.4)	367 (93.6)			
**Socio-Economic Status** (n = 1033)					
Lower	41 (5.6)	686 (94.4)	**7.61**	**0.006**	**0.51 (0.32–0.83)**
Middle & High	32 (10.5)	274 (89.5)			
**Marital status** (n = 1033)					
Married	55 (6.9)	747 (93.1)	0.24	0.63	0.87 (0.50–1.51)
Others	18 (7.8)	213 (92.2)			
**Age of Marriage** (n = 993)					
<18	34 (10.3)	295 (89.7)	**6.4**	**0.01**	**1.85 (1.14–2.99)**
≥18	39 (5.9)	625 (94.1)			
**Age of First Child Birth** (n = 937)					
≤20	46 (8.5)	497 (91.5)	2.83	0.09	1.57 (0.93–2.65)
>20	22 (5.6)	372 (94.4)			
**Parity** (n = 1033)					
≤2	31 (5.9)	495 (94.1)	2.25	0.13	0.69 (0.42–1.12)
>2	42 (8.3)	465 (91.7)			

#### Knowledge Survey

A total of 175 randomly selected women were surveyed. Less than 50% (86) of women were aware of cervical cancer (Table 3). Seventy-seven women knew of cervical cancer from one source, nine learned about the disease from more than one source, and the main source of information was friends or family. Twenty-one women (12.0%) knew two symptoms of cervical cancer, eighteen (10.2%) reported one symptom, and one woman (0.6%) knew more than three symptoms. Ten women (5.7%) knew one risk factor of cervical cancer and twenty women (11.4%) reported two risk factors. Five women (2.5%) knew one method of prevention and twenty-two women (12.6%) reported at least two preventive methods.

**Table 3:** Distribution of knowledge of Cervical Cancer (n = 175).

Survey Question	Answer Choices	n (%)

	At Least One Source (1 point)	
Heard of Cervical Cancer	Media	20 (11.4)
	Friends/family	40 (22.9)
	Medical Personal	33 (18.9)
	Do not know	89 (50.8)

	At Least Two Symptoms (2 points)	
Symptoms of Cervical Cancer	Bleeding in between periods	26 (14.9)
	Foul smell discharge	6 (3.4)
	Bleeding after intercourse in women of any age	1 (.6)
	Postmenopausal bleeding	10 (5.7)
	Urinary urgency	2 (1.1)
	Severe back ache and lower abdominal pain	16 (9.1)
	Severe swelling in one or both legs and feet	2 (1.1)
	Do not know	135 (77.1)

	At Least Two Risk Factors (2 points)	
Risk Factors of Cervical Cancer	Having multiple sexual partners	7 (4.0)
	Early sexual intercourse	8 (4.6)
	Acquiring HPV	2 (1.1)
	Cigarette smoking	8 (4.6)
	Parity and young age at first birth	3 (1.7)
	Use of oral contraceptive over 5 years	6 (3.4)
	History of sexually transmitted disease	2 (1.1)
	Poor menstrual hygiene	10 (5.7)
	Multiple pregnancies (>5)	6 (3.4)
	Do not know	145 (82.9)

	At Least Two Prevention Methods (2 points)	
Prevention of Cervical Cancer	Avoid multiple sexual partners	9 (5.1)
	Avoid early sexual intercourse	10 (5.7)
	Vaccination against HPV	7 (4.0)
	Quit smoking 80	6 (3.4)
	Avoid birth at young age	8 (4.6)
	Avoid usage of oral contraceptives	12 (6.8)
	Do not know	148 (84.6)

	At Least Two Modes (2 points)	
Treatment of Cervical Cancer	Drug therapy	40 (22.9)
	Radiotherapy	13 (7.4)
	Surgery	27 (15.4)
	Do not know	125 (70.9)

	At Least One Test (1 Point)	
Screening Tests of Cervical Cancer	VIA	4 (2.3)
	VILI	4 (2.3)
	Pap smear	23 (13.1)
	Do not know	143 (81.7)
	Others (blood test)	1 (0.6)

	At Least One Eligibility Criteria (1 Point)	
Eligibility Criteria for Screening	Women age of 25 years and above	64 (36.6)
	Women having multiple sex partners	6 (3.4)
	Elderly women	20 (11.4)
	Do not know	84 (48.0)
	Others (>18years)	1 (0.6)

	At Least One Location (1 point)	
Location of Screening	Government Centers	80 (45.7)
	Private Centers	165 (94.3)
	Do not know	24 (13.7)

	At Least One Frequency Criteria (1 point)	
Frequency of Screening	Once every year	27 (15.4)
	Once every 3 years	14 (8.0)
	Once every 5 years	1 (0.6)
	Do not know and others	133 (75.4)

	At Least 2 Points	
Knowledge of HPV	Heard of HPV Vaccine	1 (0.6)

With regard to treatment for cervical cancer, twenty-one women (12%) reported one treatment and twenty-nine (16.6%) knew two treatment options. Thirty-one women (17.7%) knew at least one test for cervical cancer screening. Eighty-one (46%) knew at least one location to be screened for cervical cancer and seventy (40%) knew of two or more locations. Ninety (51.4%) women reported at least one eligibility criteria for screening and fifteen (8.6%) knew the frequency of screening. One woman, a nurse, had heard of the HPV vaccination. The distribution of responses to the knowledge regarding cervical cancer is shown in Table 3.

Among all respondents, 148 (84.6%) had poor knowledge, 18 (10.3%) had moderate knowledge, and only 9 (5.1%) had good knowledge of cervical cancer. Knowledge scores ranged from zero to thirteen points with a mean (SD) knowledge score of 3.47 (3.4) points and a median of 2 points.

Women aged 41 or less had better knowledge as compared to women above 41 years (p-value = 0.009, OR = 0.31 (0.12–0.77). There was no significant association of knowledge with religion, education, occupation, socioeconomic status, marital status, parity, age of marriage or age of first childbirth.

## Discussion

Among the women ages 25–65 living in five low income areas in Vellore, Tamil Nadu, we found that only 7.1% had undergone cervical cancer screening at least once in their lifetime. Additionally, less than 15% of women had a pelvic exam. Almost 85% of the 175 surveyed women had poor knowledge of cervical cancer and less than 25% knew of symptoms, risk factors, or preventative measures for cervical cancer. No women in our study received the HPV vaccine and almost no women knew of the vaccination. Our results provide the first description of the current state of screening and vaccination for women who belong to the communities that receive health care from our unit’s outreach clinics.

We found low levels of primary prevention via the HPV vaccine. Although the HPV vaccination was introduced in India in 2008, it has yet to be included in the immunization program in India and no women in our study received the vaccine [11]. Several barriers exist with regard to the inclusion of the HPV vaccine in the government program. Public concern regarding the vaccine arose from the deaths of seven girls who received the vaccine [11,13]. Although subsequent investigations concluded that these deaths were not linked to vaccination, the vaccination campaign was never restarted [12]. Additionally, the high cost of the HPV vaccine prevents women from receiving the vaccination. The women included in our study are beyond the target age for the HPV vaccination; therefore, screening to detect and treat precancerous lesions remains the only form of prevention available to them.

Despite this, few women in our community are currently screened for cervical cancer. Past reports suggest screening prevalence is highly variable across states and within districts. In Tamil Nadu, the average prevalence of screening was found to be 31.0% (29.7–32.4) among women ages 30–49 [6]. Our population, however, reported even lower levels of lifetime screening. Further abetting this gap in care, women’s knowledge of cervical cancer in our population was lower than other studies in India [9,13].

We suspect that multiple factors may affect women’s ability and desire to participate in screening. The majority of women were willing to be screened for cervical cancer and knew of locations for screening, but most of the women did not know about the screening tests. Screening is a preventative service, which is not a priority for asymptomatic and low-income people who are struggling with more acute day-to-day problems [14,15]. Further, our communities lack public transportation, and so women would need to travel 2.5–3 km for screening. Once women reach the main clinic, they experience long wait times to be seen by a healthcare professional. Finally, although the cost of care is subsidized at the urban health center, patients must still pay a fee for screening. In our communities, the majority of women are homemakers, and depend financially on their husbands. Their husbands typically decide whether to finance screening, creating an additional barrier for women to access this preventative service. Women of a higher socioeconomic status, however, were more likely to be screened—suggesting that women may be more likely to seek screening if the financial barrier was removed. Overall, the fundamental lack of understanding of prevention combined with economic and time constraints, and an overwhelming lack of knowledge about cervical cancer, prevents women from being screened.

Our study has some limitations. First, the survey to assess women’s knowledge was conducted by a female research assistant and a male translator. It is possible that women were uncomfortable discussing gynecologic issues in the presence of a man. The research assistant conducted the survey, but there is a possibility that some information was not correctly conveyed to the survey participant. Finally, women self-reported their age, age of marriage, and age of first child, but some women may not know these ages, and so the reported ages may not be accurate. Despite these limitations, we believe that we were able to gain unique access to this population due to the active engagement and ongoing relationships between the community and community health workers.

Given women’s willingness for screening and the active participation of health care workers in their community, there is an opportunity to enhance community level awareness and streamline screening processes. First, we can create awareness through community-based education programs conducted by the community health workers in each community. Additionally, once community health workers refer women for screening, we can facilitate screening by reducing wait times once women are at the clinic. For example, we could establish pre-determined days during which a room in the urban health center is dedicated to screening. Increasing awareness and reducing barriers will hopefully improve the prevalence of screening amongst our women.

## Declaration

All authors had full access to of the data in the study. Ms. Reichheld and Dr. Ruby take responsibility for the integrity of the data and accuracy of the data analysis. All authors were responsible for the design of the study. Ms. Reichheld was responsible for the collection and management of the data. All authors were involved in the review and approval of the manuscript.
